# CLASH Analyst: A Web Server to Identify In Vivo RNA–RNA Interactions from CLASH Data

**DOI:** 10.3390/ncrna8010006

**Published:** 2022-01-12

**Authors:** Wei-Sheng Wu, Jordan S. Brown, Pin-Hao Chen, Sheng-Cian Shiue, Dong-En Lee, Heng-Chi Lee

**Affiliations:** 1 Department of Electrical Engineering, National Cheng Kung University, Tainan 701, Taiwan; wessonwu@mail.ncku.edu.tw (W.-S.W.); bba753951@gmail.com (P.-H.C.); t50504t@gmail.com (S.-C.S.); ttyy66995@gmail.com (D.-E.L.); 2 Department of Molecular Genetics and Cell Biology, University of Chicago, Chicago, IL 60637, USA; jordanbrown@uchicago.edu

**Keywords:** non-coding RNA, RNA–RNA interactions, CLASH, miRNA targets, piRNA targets

## Abstract

Non-coding RNAs, such as miRNAs and piRNAs, play critical roles in gene regulation through base-pairing interactions with their target molecules. The recent development of the crosslinking, ligation, and sequencing of hybrids (CLASH) method has allowed scientists to map transcriptome-wide RNA–RNA interactions by identifying chimeric reads consisting of fragments from regulatory RNAs and their targets. However, analyzing CLASH data requires scientists to use advanced bioinformatics, and currently available tools are limited for users with little bioinformatic experience. In addition, many published CLASH studies do not show the full scope of RNA–RNA interactions that were captured, highlighting the importance of reanalyzing published data. Here, we present CLASH Analyst, a web server that can analyze raw CLASH data within a fully customizable and easy-to-use interface. CLASH Analyst accepts raw CLASH data as input and identifies the RNA chimeras containing the regulatory and target RNAs according to the user’s interest. Detailed annotation of the captured RNA–RNA interactions is then presented for the user to visualize within the server or download for further analysis. We demonstrate that CLASH Analyst can identify miRNA- and piRNA-targeting sites reported from published CLASH data and should be applicable to analyze other RNA–RNA interactions. CLASH Analyst is freely available for academic use.

## 1. Introduction

RNA molecules perform some of the most fundamental and important functions in organisms, including coding for proteins (mRNAs), providing structural support (tRNAs, lncRNAs), acting as vital enzymes (snRNAs, ribozymes), and guiding proteins to regulate other RNAs (miRNAs, piRNAs, siRNAs). Although discovered only relatively recently, this last category of RNA function has been shown to play a prominent role in posttranscriptional and transcriptional gene regulation. By guiding Argonaute family proteins to RNA targets using base-pairing interactions, miRNAs, piRNAs, and siRNAs can effectively and specifically target diverse mRNAs for regulation. For example, it has been estimated that ~50% of human genes are regulated by miRNA [1,2,3]. To attempt to identify functionally significant RNA–RNA interactions, researchers have historically relied on bioinformatic prediction to locate possible regulatory events based on sequence complementarity, sequence conservation of target sites, and by experimentally validated results of each regulatory site individually [4].

**Figure 1:**
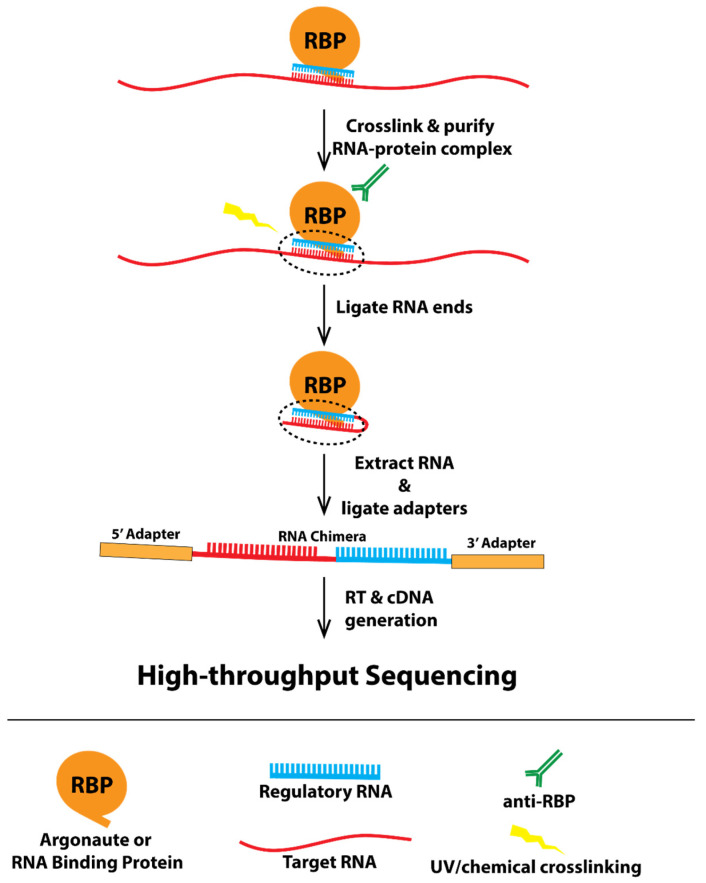
A model showing the experimental framework underlying CLASH.

Recently, a technique has been developed to capture in vivo RNA–RNA interactions, called CLASH (crosslinking, ligation, and sequencing of hybrids) [5,6,7]. UV crosslinking and immunoprecipitation (CLIP) is a powerful biochemical approach to map protein-RNA interactions in vivo [8]. CLASH builds off of the idea of capturing RNA–protein complexes in vivo, as developed with CLIP, and adds a ligation step to produce chimeric RNA molecules made up of the regulatory RNA, which was held by the protein and the target RNA in proximity to regulatory RNAs in the cell (Figure 1, a model showing how CLASH works). In addition, several other techniques, such as iCLIP and CLEAR-CLIP [9,10], also generate similar chimeric RNA reads that reveal in vivo RNA–RNA interactions. Although CLASH and other CLASH-like data have proven powerful in identifying various types of RNA–RNA interactions, including miRNAs, piRNAs, and other non-coding RNAs with their targets, the data are not trivial to analyze. Sequenced CLASH reads represent fusions of two different RNA molecules that cannot be mapped using traditional approaches as accomplished with RNA-seq. Furthermore, currently developed chimera-identifying programs such as CLAN and Hyb are limited in their accessibility to researchers with little bioinformatic knowledge [11,12]. CLAN identifies RNA–RNA pairs from chimeric reads but does not integrate other critical components of the analyses, including preprocessing of sequencing reads and postprocessing analyses such as calculation of base-pairing energy, leaving these necessary steps up to the user. Hyb is a command-line program that requires some degree of computational experience to use. Furthermore, neither of these tools include a visual display of these RNA–RNA interactions, nor do they have the capability to search for sites with specific criteria.

To allow scientists with and without knowledge of bioinformatics to perform integrative analysis of CLASH data, we developed CLASH Analyst, which accepts raw CLASH data and outputs fully processed and easy-to-understand representations of the transcriptome-wide RNA–RNA interactions contained within those data. CLASH Analyst allows users to select default or customized parameters of the analysis pipeline, which are comprehensively explained within the application, to parse their data according to their specific needs. Furthermore, CLASH Analyst’s output displays comprehensive information about each identified RNA–RNA interaction, including the sequences of the regulatory RNA and its target, the relative abundance and location of that interaction, and the free energy of pairing for the targeting event. All resultant analyses are available for user-friendly browsing within the CLASH Analyst interface, as well as for download, to facilitate any downstream analyses.

During the development of CLASH Analyst, another tool similar to CLASH Analyst named ChiRA was published that provides a similar analysis pipeline to analyze RNA–RNA interactions [13]. While ChiRA can also perform comprehensive analyses on RNA–RNA interaction data, it was provided as an analysis tool in Galaxy and, therefore, requires users to be familiar with the Galaxy platform [14]. On the contrary, CLASH Analyst is a completely standalone web server that allows users to customize their search criteria by choosing searching algorithms and adjusting parameters, which is more intuitive to a user unfamiliar with the Galaxy platform.

## 2. Webtool Description

### 2.1. General Framework

**Figure 2:**
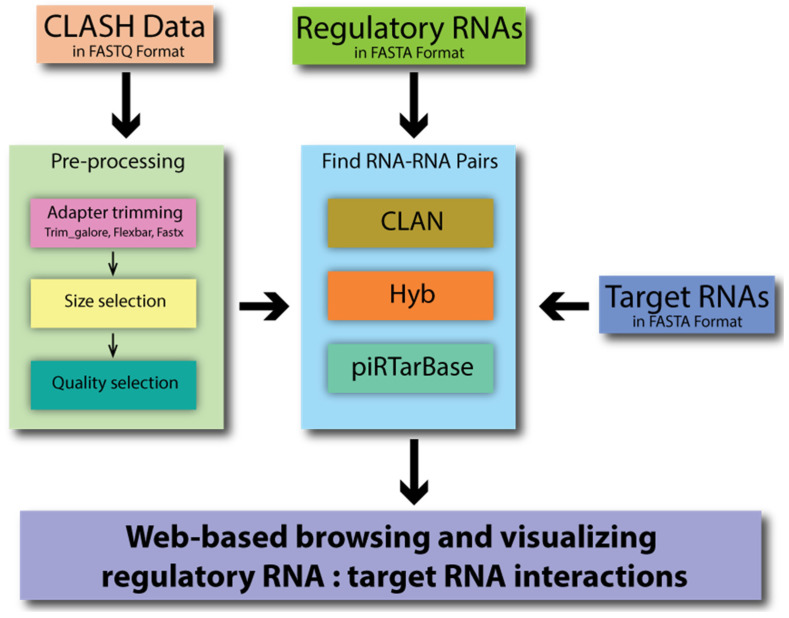
A graphical depiction of CLASH Analyst’s workflow.

CLASH Analyst is a tool that identifies RNA–RNA interactions using user-provided raw CLASH data in three simple steps (Figure 2). First, the user will upload three files: CLASH raw data, a file containing the presumed regulatory RNA sequences, and a file containing the presumed target RNA sequences (Figure S1A). Second, the user will choose to apply default or customized parameters to preprocess the raw sequencing reads, which removes RNA linker sequences and filters the sequenced reads based on desired sequencing quality. Third, the user can choose how CLASH Analyst will search for chimeric reads using a specific searching algorithm, such as pirTarBase, CLAN, or Hyb [11,12,15] (Figure S1B). A Job ID and a link are provided upon job submission to access the results of the analyses. As the process takes some time to run, users can provide an email address so that they can be informed once the analysis is complete. Upon completing the analysis, the CLASH Analyst will provide two summary tables that indicate the parameters chosen for the search and the numbers of chimera and RNA–RNA interactions found (Figure S1C). CLASH Analyst allows users to browse or search through the identified chimeric reads according to the names of their input regulatory RNAs, the names of their target RNAs, or by RNA–RNA pair according to the nature of the chimeric reads (abundance or pairing energy). For example, if users are interested in identifying the targets of a given regulatory RNA (such as *lin-4* miRNA), they can immediately sort all chimeric reads containing such RNA of interest, based on the abundance of the interaction in chimeric read count (Figure S1D). For each RNA–RNA interaction, such as *lin-4* miRNA and its target *lin-14* (T25C12.1a.1), the user can click to reveal comprehensive information of each chimeric read, including where along each molecule the chimeric reads originated, how many reads were captured, and where mismatches occurred within the base-pairing interaction (Figure S1E). The user can also download the complete analysis results.

### 2.2. Data Input

The user must upload a compressed FASTQ file and two compressed FASTA files (Figure S1A). The FASTQ file contains the raw data from the CLASH experiment that the user wishes to analyze. The first compressed FASTA file must correspond to the regulatory RNA sequences, and the second FASTA file should correspond to all RNA sequences the user wants CLASH Analyst to query as the target sequences. Example uploaded files are included and can be loaded by clicking “Load example files”. In addition, common regulatory RNA sequences (e.g., human miRNAs, *C.elegans* miRNAs, *C.elegans* piRNAs) and target RNA sequences (e.g., human mRNAs, *C.elegans* mRNAs) are preloaded and can be selected by the user using a drop-down menu to skip uploading/downloading those data.

### 2.3. CLASH Read Preprocessing

CLASH Analyst performs all necessary steps to preprocess the raw reads (including adaptor/barcode sequence removal and reads quality control, etc.) so that chimeras can be identified. The user can choose default settings or select specific settings according to how the CLASH libraries were prepared (Figure S1B). Users should provide adaptor information to allow precise trimming of adaptor sequences from the sequencing reads. Some library preparations include the ligation of a multiplexing barcode to the 5′ end of each RNA. This sequence should be provided so that CLASH Analyst can trim it from reads before downstream analysis. Some library preparations may also include some random nucleotides in the 5′ adaptor to act as unique molecular identifiers (UMIs). In this case, the user should provide the number of random nucleotides included in the 5′ adaptor to allow CLASH Analyst to trim UMIs. For example, if the 5′ adaptor includes 6 random nucleotides, then the user should input NNNNNN as part of the 5′ barcode input. Similarly, the 3′ adaptor sequence can be provided for precise trimming. Since Trim galore is capable of detecting 3′ adaptor sequences without prompting (although providing the sequence can help), Trim galore is chosen as the default trimming tool [16]. If the user chooses this tool, then 3′ adaptor sequence input is optional. The user can also select from two other tools, flexbar or fastx, to perform adaptor trimming [17,18]. In addition, the user provides the range of RNA sizes that will be analyzed. Based on how molecules were selected during library construction, the user should have an expectation of the length of reads following adaptor trimming and select/filter only reads of a certain length range for analyses. For example, if RNA molecules between 17 nt and 70 nt long were selected and ligated to adaptors, then the trimmed reads should be 17–70 nt in length. Finally, the user can filter reads that do not meet a quality threshold, which is encoded in the FASTQ file called the Phred score. Phred scores are assigned during sequencing based on the probability of errant base calling. The default threshold for the Phred score is 30. A Phred score of 30, 40, or 50 for a particular base corresponds to a 99.9%, 99.9%, or 99.999% likelihood that the called base is accurate, respectively. CLASH Analyst can also accept data that have been preprocessed prior to being uploaded if the user wishes to perform these steps themselves.

### 2.4. Identify Chimeras

Once data are preprocessed, CLASH Analyst identifies chimeric reads using default or user-defined settings. The user can choose among three different algorithms to assign chimeric reads: CLAN [11], Hyb [12], and piRTarBase [15] (Figure S1C). These algorithms differ on how they search for chimeric reads, resulting in some differences in the set of interactions they each identify (details can be found in “Section 3. Analysis Results”). The default searching tool in CLASH Analyst is CLAN.

If the user chooses to use CLAN, they can use default settings or specify criteria for searching chimeric reads, including (1) the minimum size of each fragment within a chimeric read for analyses; (2) how much overlap between the two fragments is allowed (if the default value of 4 is selected, then 4 nt would be allowed to map to each fragment simultaneously); (3) how many hits will be allowed for each fragment, as each fragment could match multiple user-input FASTA sequences. Similarly, the user can use default or specify criteria for searching chimeric reads using Hyb or piRTarBase. We recommend users first analyze their data with CLAN if they are unsure of which algorithm to choose, as CLAN performs the least stringent analysis and identifies more RNA–RNA interaction candidates (for details, see Section 3). Then, to further refine the results, users can choose to rerun their data using piRTarBase or Hyb. To reanalyze data using a different algorithm quickly, the user can select the “Reanalyze old data?” option and adjust the algorithm settings accordingly after providing a Job ID (provided upon original submission). Users can access analysis results using the provided Job ID for 30 days. Extensions to this 30-day default can be provided upon reasonable request.

### 2.5. User Information

Once the user has uploaded all data and selected all necessary settings, they can submit the analysis job by pressing submit at the bottom of the page. A Job ID and a website link are provided for the users to check the status and examine the results. The analysis can typically take several hours to run (sometimes a day for larger datasets), so we provided an option to input an email address for the user to be notified once the analysis is complete. This is an optional, step and the email address is used only to notify and send the user a link to the results page.

### 2.6. Chimeric Reads Output

The user can input the Job ID or use the website link to browse the results within CLASH Analyst. The user can choose one of the three ways to display their results, which include browsing by (1) regulatory RNAs within the chimera, (2) target RNAs within the chimera, or (3) individual RNA–RNA pair ranked by chimera abundance or pairing energy. Regardless of what the user chooses to browse by, a summary of the analysis is first displayed in two tables (Figure S1D). The first shows all the user-specified settings that were used to perform the analysis, while the second shows the number of various reads identified by CLASH Analyst, including (1) the number of reads provided by the user, (2) the number of reads and unique reads after trimming the adaptors, (3) the number of those trimmed reads that were identified as chimeric, and (4) the number of RNA–RNA interactions identified. The browsable and searchable table containing the remainder of the output will depend on which browse mode was selected and will be discussed separately here. By selecting any of these three options, users can also download the full output files organized by CLASH read, regulatory RNA name, target RNA name, and RNA–RNA interaction in .csv format.

#### 2.6.1. Browse by Regulatory RNA Name

The browsable and searchable output table will contain three columns: A column corresponding to regulatory RNA names, a column displaying the number of identified targets, and an option to show more details about the targets for each regulatory RNA (Figure S1D). The user can choose to show more details for any regulatory RNA that was included in the input. This reveals all target RNA sequences identified within chimeras containing the selected regulatory RNA in a tabular format. Within the table, users can find the number of chimeric reads representing the number of unique chimeras containing that RNA–RNA interaction, such as *lin-4* and *lin-14 (T25C12.1a.1*). Additionally, any of the regulatory RNA–target RNA pairs can be further examined by clicking “show interaction”. A graphic showing the interactions between target RNA and regulatory RNA is available for viewing (Figure S1E). Additionally, a table that shows each identified interaction by chimeric read can be viewed. This table shows information about each hybrid, including the number of reads (read count), the thermodynamic favorability of the identified regulatory RNA and target RNA hybrid, where the interacting site mapped, and the predicted base pairing between the regulatory RNA and its target RNA.

#### 2.6.2. Browse by Target RNA Name

The output table contains four columns: A column corresponding to the target RNA name, a column displaying the number of unique chimeras that contained that target RNA, a column displaying the number of unique regulatory RNAs found to pair with that target, and an option to show interactions involving each target RNA (Figure S1D). By clicking “show interaction”, the user can view a detailed graphic and table outlining all interactions involving that target RNA. The graphic and table contain the same information outlined at the end of the Browse by Regulatory RNA Name section.

#### 2.6.3. Browse by RNA–RNA Pair

Users can also browse by RNA–RNA pair. The results can either be sorted according to read count or according to the most favorable binding energy (Figure S1D). The output table has rows corresponding to each unique chimera and presents information about each hybrid, including the number of reads (read count), the thermodynamic favorability of the identified regulatory RNA and target RNA hybrid, where the interacting site was mapped, and the predicted base pairing between the regulatory RNA and its target RNA. The webpage only shows the first 10,000 results. To view the rest, the user can download the result table in .csv format, as discussed above.

## 3. Analysis Results

**Figure 3:**
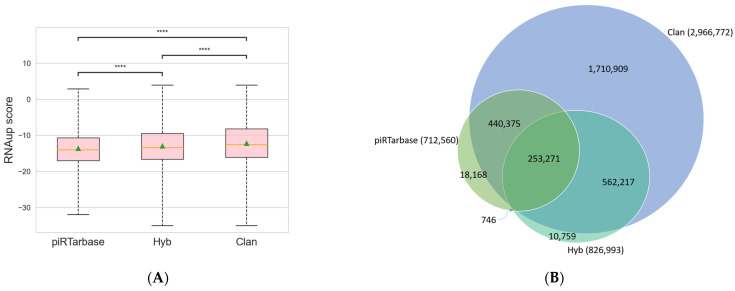
Comparison of RNA–RNA interactions identified from three available searching algorithms in CLASH Analyst: (**A**) the distribution of RNAup scores from RNA–RNA interactions identified in three searching algorithms. The lower the RNAup score, the more thermodynamic favorable interactions are predicted. ****: *p* < 0.0001; (**B**) a Venn diagram showing the unique and overlapping RNA–RNA interactions identified from three searching algorithms.

Among the three searching algorithms available in CLASH Analyst, piRTarBase is the most stringent, and CLAN is the least stringent but most sensitive algorithm. Specifically, piRTarBase first searches for the regulatory RNA sequences within each read, and the default settings do not tolerate mismatches or deletions. It then uses the remaining sequence to identify targets with perfect matches. By using its default setting, piRTarBase requires the presence of intact regulatory RNA and does not allow insertions between regulatory RNA and target RNAs. Hyb tolerates short deletions and insertions in regulatory RNAs and target RNAs, and it also tolerates overlapping fragments. Nonetheless, Hyb will then choose one RNA–RNA interaction with the highest score among multiple possible matches, which increases its searching stringency. The least stringent algorithm, CLAN, will identify more interactions using more sensitive searching and will report all possible matches, resulting in multiple interpretations of RNA–RNA interactions for some individual chimeric reads. To compare the performance of each of the three searching algorithms (CLAN, Hyb, and piRTarBase) [11,12,15] available in CLASH Analyst, we used a previously published *C.elegans* PRG-1 CLASH dataset (SRR6512652) [7] and compared the outputs of these three algorithms. We identified 712,560 RNA–RNA interactions from this dataset by piRTarBase, compared with 826,993 identified by Hyb and 2,966,772 identified by CLAN. We found that using the more stringent options (piRTarBase and Hyb), the identified chimeras generally have more favorable free energies of hybridization (Figure 3A), which suggests that they are more likely to represent true in vivo interactions [19]. In these analyses, CLAN can identify the majority of interactions identified by piRTarBase or Hyb and report many more possible interactions (Figure 3B), although some will represent lower confidence hits.

To compare CLASH Analyst’s performance with previously analyzed results, we used the default parameter setting of CLASH Analyst to analyze three CLASH datasets including human miRNAs, *C. elegans* miRNAs, and *C. elegans* piRNAs [6,7,9]. Since previous CLASH studies of *C. elegans* miRNAs and piRNAs did not provide the full details of their results, we were not able to compare the number of targets found in CLASH Analyst to those from previous studies. Nonetheless, we found that *C. elegans* miRNA-binding sites are enriched at 3′ untranslated regions (UTRs) of their mRNAs, consistent with the previous report (Figure S2) [9]. In addition, we found that regulatory RNA-binding sites identified by CLASH Analyst include all the key examples shown in the original CLASH studies, such as the interactions between *lin-4* miRNA and *lin-14* mRNA, as well as *21ur-1 (type 2)* piRNA and *xol-1* mRNA (Figure S3). In addition, using a human miRNA CLASH dataset (SRR959751), we found that the default searching algorithm (CLAN) in CLASH Analyst identified significantly more miRNA–target interactions (13,860 vs. 1436) than using the Hyb searching algorithm, which was used to identify RNA–RNA interactions in the previous report (Figure S4) [6]. Another recently published tool, ChiRA, was reported to identify approximately fourfold more interactions than the original human miRNA CLASH mRNA–target interactions [13]. Since ChiRA groups RNA–RNA interactions with similar interactions into common reads loci (CRLs), it is difficult to directly compare the results of ChiRA to CLASH Analyst. Taken together, we conclude that CLASH Analyst can identify similar or more miRNA targets than the currently published methods.

## 4. Discussion

CLASH is a critical approach to identify RNA–RNA interactions, including the interaction between small non-coding RNAs and their targets. However, the analyses of CLASH data are not trivial, and current tools are limited to researchers with advanced bioinformatic experience. Our goal in developing the CLASH Analyst was to allow researchers of varying levels of bioinformatics expertise to harness the power of CLASH to identify RNA–RNA interactions easily and quickly. CLASH Analyst can take raw sequencing reads and perform comprehensive analysis on CLASH data. CLASH Analyst further provides browser and virtualization tools that are critical for researchers to identify RNA–RNA interactions of interest.

By analyzing published CLASH datasets using three available searching algorithms offered by CLASH Analyst, we showed that CLAN identified more RNA–RNA interactions, while pirTarbase identified high confidence interactions whose pairing are generally thermodynamically more stable. Since CLASH Analyst offers three searching algorithms with different stringencies, it empowers its users to perform customized searching by choosing the searching algorithm and selecting specific parameters based on their preferences and needs. In addition, we found that by repeating analysis of published CLASH datasets using CLASH Analyst, we could identify as many or more RNA–RNA interactions as reported in the respective original studies. We also confirmed that we could readily identify all RNA–RNA interaction signatures relevant to the respective studies, such as 3′ UTR enrichment for miRNA-targeting events and physiologically relevant miRNA and piRNA-targeting events. During the development of CLASH Analyst, our analyses of piRNA CLASH datasets revealed genome-wide, piRNA-binding sites in *C. elegans* [15].

In addition to exploring RNA–RNA interactions from user-generated CLASH datasets, CLASH Analyst will be valuable for researchers of diverse fields to identify RNA–RNA interactions of interest from published CLASH datasets. Many published CLASH studies highlight specific aspects of the RNA–RNA interactions. The majority of RNA–RNA interactions were not mentioned due to space constraints or lack of interest. Although some of these papers include supplemental material that contains the processed results from the CLASH experiment, the list of RNA–RNA interactions is not exhaustive and may not include specific details of the interactions that are critical for further analyses. We envision CLASH Analyst as the perfect tool for researchers to retread previously published data, to find unappreciated results that can be used to support or formulate new hypotheses. In addition, CLASH Analyst can be used to identify noncanonical RNA–RNA interactions. For example, it has been proposed that Argonaute proteins can bind tRNA fragments (i.e., tRFs) to translationally repress mRNAs [20]. Researchers interested in this hypothesis can search through publicly available Argonaute CLASH datasets from various organisms to find evidence of tRF–mRNA interactions using CLASH Analyst. As RNA–RNA interactions have proven to be increasingly present in diverse biological contexts, novel techniques have been developed to capture these interactions. Although CLASH Analyst was developed to assist researchers in finding interactions from CLASH data, we anticipate our tool to be equally as useful in identifying RNA–RNA interactions in datasets from other methods which generate chimeric reads, such as recently developed techniques MARIO and RIC-seq [21,22]. Therefore, CLASH Analyst will lower the barrier for researchers who are interested in employing these exciting new tools but reticent due to their inexperience with advanced bioinformatics.

We are confident that CLASH Analyst will allow users from any background to analyze their own data or previously published data to identify potential in vivo RNA–RNA interactions. We believe that with such advanced bioinformatic techniques available to all researchers in the RNA community, CLASH Analyst will allow for significant and novel advancement in our understanding of RNA regulation.

## 5. Materials and Methods

CLASH Analyst analysis pipeline behind the web interface was implemented using awk, bash, and Python scripts. The web interface of CLASH Analyst was constructed using Django, a Python web framework that encourages rapid web development. All figures in CLASH Analyst were generated using D3.js, a JavaScript library that provides powerful visualization components. All tables in CLASH Analyst were generated using DataTables, a table enhancing plug-in for the jQuery JavaScript library, which adds sorting, paging, and filtering abilities to plain HTML tables with minimal effort. CLASH Analyst is available at https://cosbi7.ee.ncku.edu.tw/CLASHanalyst/ (main site) and https://cosbi.ee.ncku.edu.tw/CLASHanalyst/ (backup site).

## Data Availability

CLASH Analyst is freely available for academic use at https://cosbi7.ee.ncku.edu.tw/CLASHanalyst or https://cosbi.ee.ncku.edu.tw/CLASHanalyst (backup site). The source code of CLASH Analyst can be downloaded from Github at https://github.com/t50504/CLASHanalyst.

## Supplementary Materials

The following are available online at https://www.mdpi.com/article/10.3390/ncrna8010006/s1, Figure S1: A step-by-step depiction of the CLASH Analyst workflow, Figure S2: The distribution of miRNA and piRNA-binding sites in *C. elegans*, Figure S3: Examples of RNA–RNA interactions identified by CLASH Analyst, Figure S4: A comparison of RNA–RNA interactions identified by CLAN and Hyb.
