# Supplementary material for: CLASH Analyst: A Web Server to Identify In Vivo RNA–RNA Interactions from CLASH Data
Source: Noncoding RNA. 2022 Jan 12;8(1):6. doi: 10.3390/ncrna8010006 (PMC8788457; doi:10.3390/ncrna8010006)
Supplement: Supplementary file 1 [file ncrna-08-00006-s001.zip › ncrna-1534562-supplementary.pdf]

Supplementary information for:

CLASH Analyst: a webserver to identify *in vivo* RNA-RNA interactions from CLASH data

Wei-Sheng Wu, Jordan S Brown, Pin-Hao Chen, Sheng-Cian Shiue, Dong-En Lee, Heng-Chi Lee

This supplementary information includes:

Figure S1. A step-by-step depiction of the CLASH Analyst Workflow

Figure S2. The distribution of miRNA and piRNA binding sites in *C. elegans*.

Figure S3. Examples of miRNA and piRNA targets identified by CLASH Analyst

Figure S4. A comparison of RNA-RNA interactions identified by CLAN and Hyb

Tutorials for using CLASH Analyst

CLASH Analyst webserver construction

Browser compatibility

References cited in supplementary information

Figure S1. A step-by Step depiction of the CLASH Analyst Workflow

Figure S1A (top) & S1B (bottom)

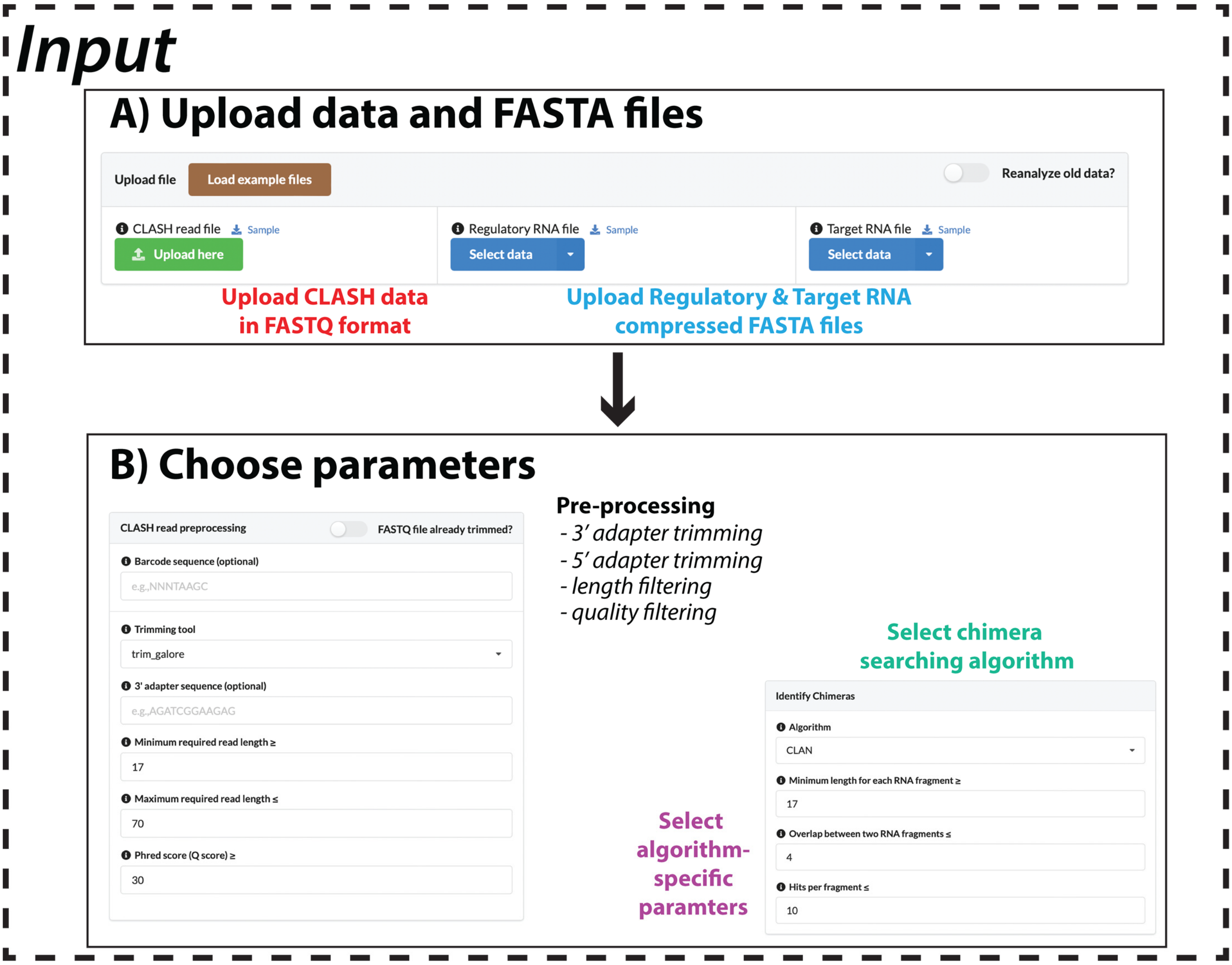

Figure S1 (continued).

Figure S1C (left) & S1D (right)

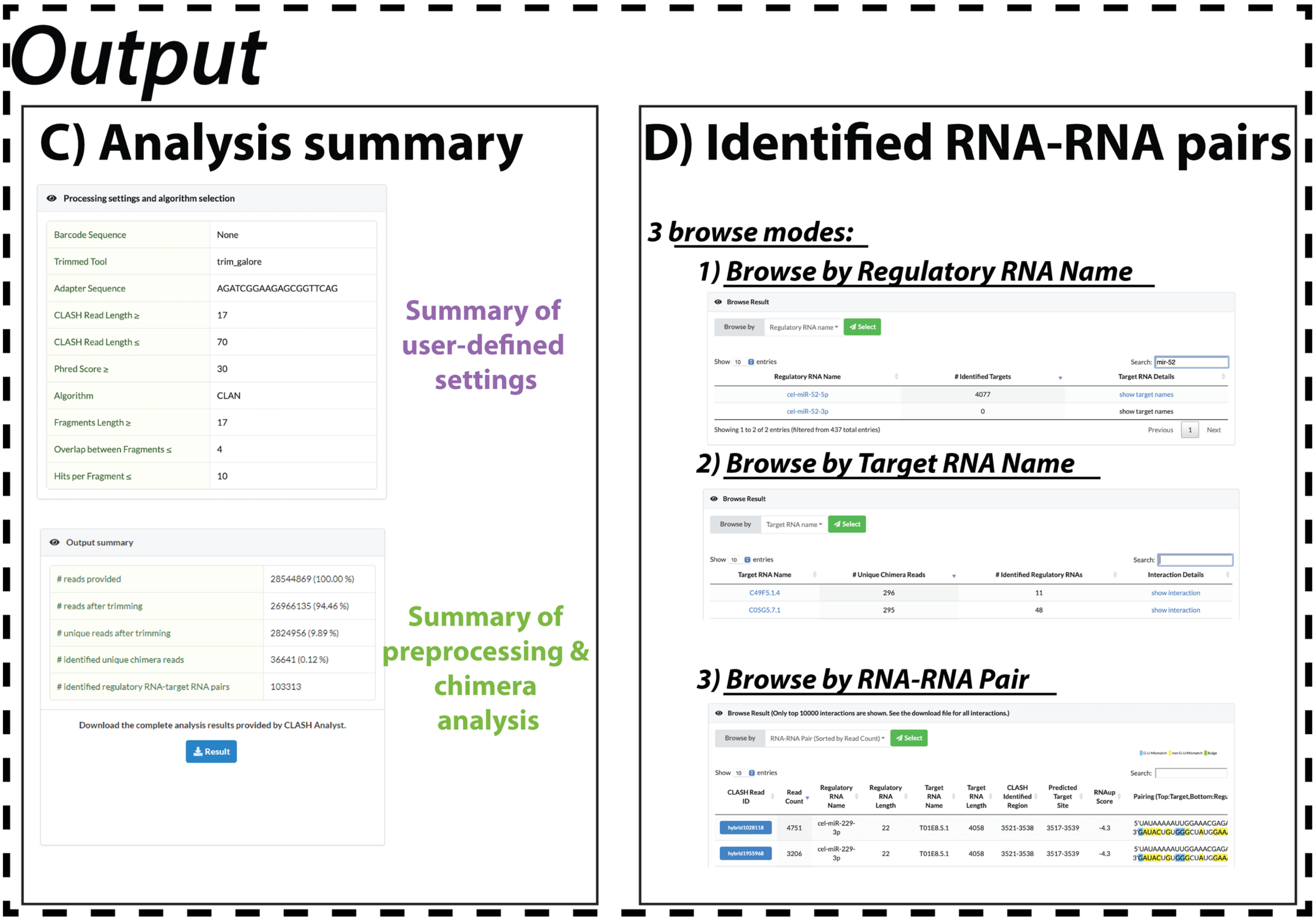

Figure S1 (continued).

Figure S1E

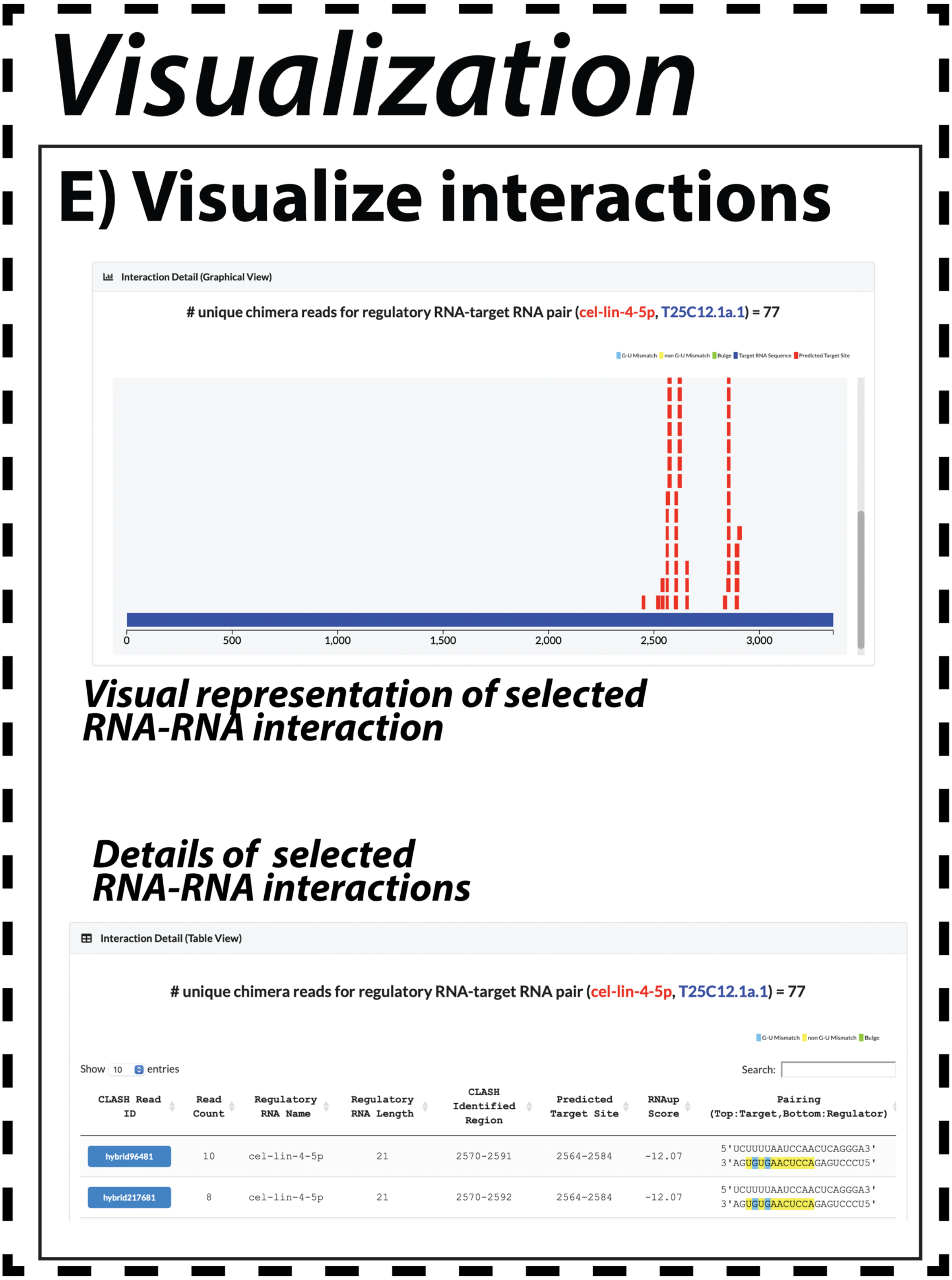

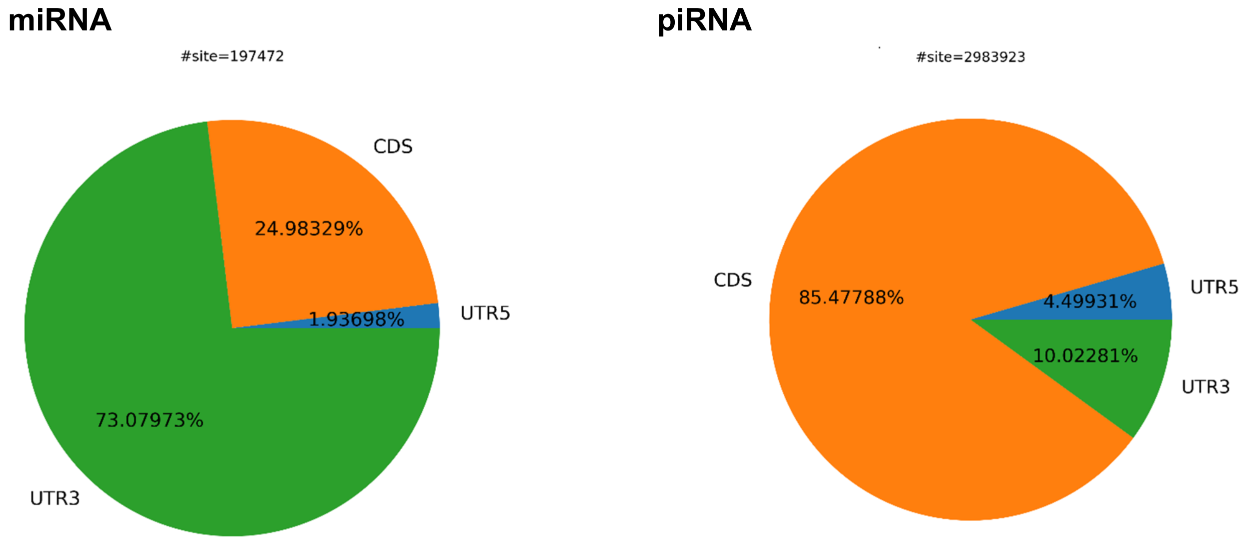

Figure S2. The distribution of miRNA and piRNA binding sites in *C. elegans*. Note that while more piRNA targeting sites are found in CDSs, more miRNA targeting sites are found at 3' UTRs.

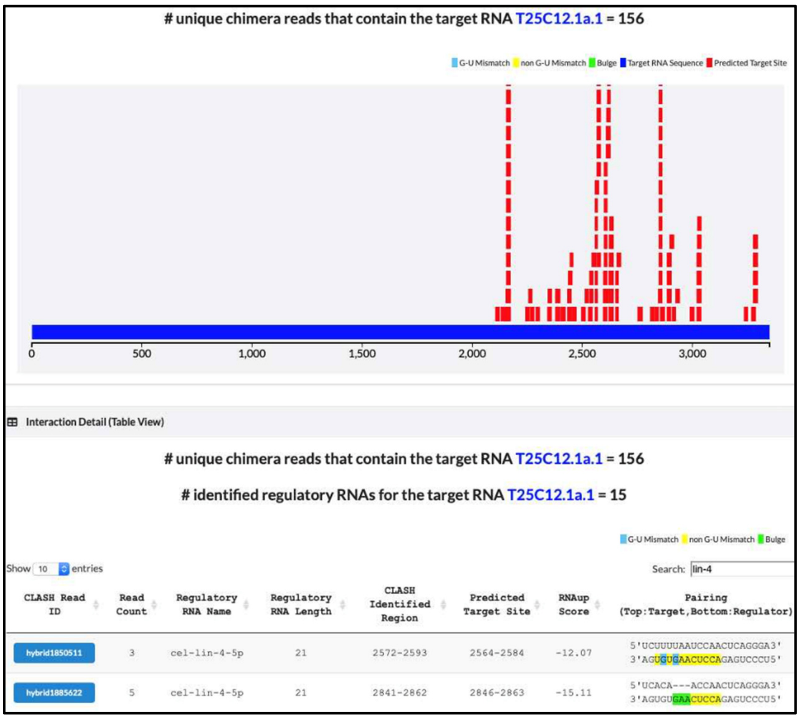

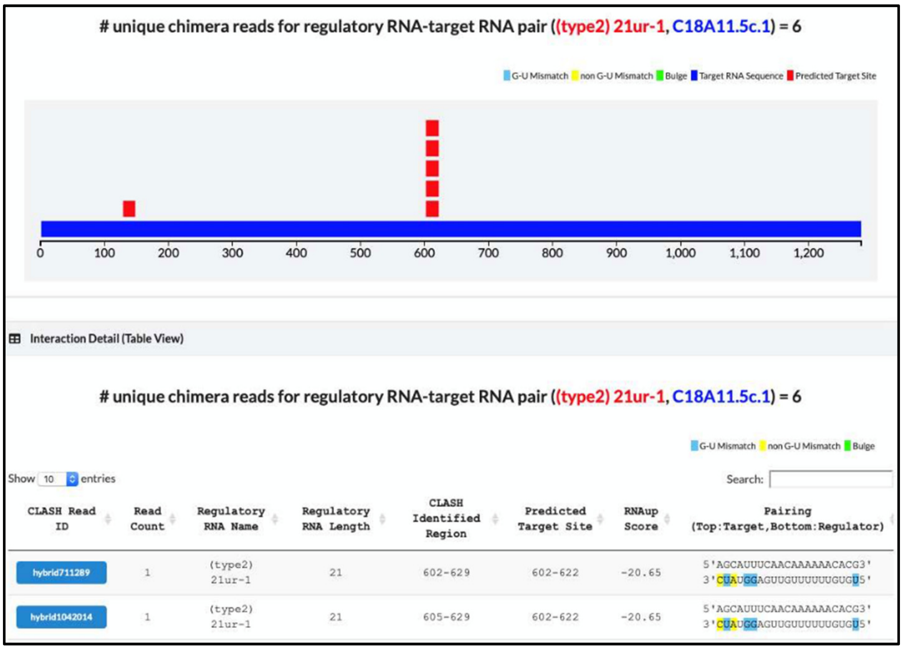

Figure S3. Examples of RNA-RNA interactions identified by CLASH Analyst

The RNA-RNA interactions identified by CLASH Analyst, including *lin-4* miRNA and *lin-14* (T25C12.1a.1) mRNA (top), and 21u-x1 piRNA and xol-1 (C18A11.5c.1) mRNA (bottom).

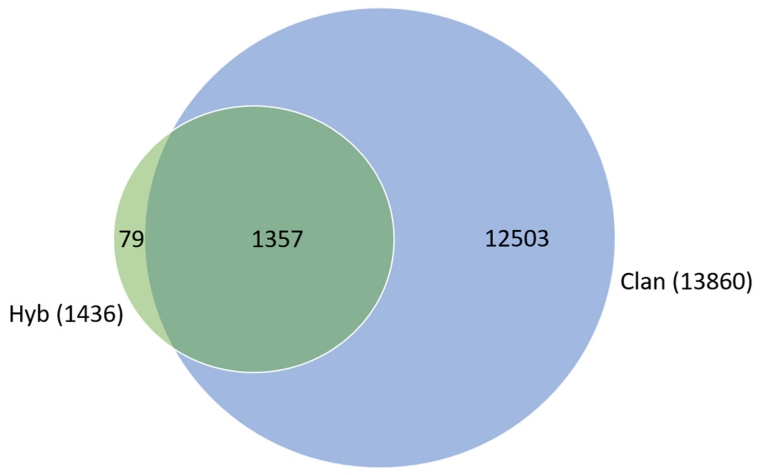

Figure S4. A comparison of RNA-RNA interactions identified by CLAN or Hyb.

A Venn diagram showing the unique and overlapping RNA-RNA interactions identified by CLAN or Hyb in analyzing the human miRNA CLASH data set (SRR959751).

Tutorials for using CLASH Analyst

Input

The user must upload a compressed FASTQ file, and two compressed FASTA files (Figure S1A). The FASTQ file will be the raw data from the CLASH experiment that the user wishes to analyze.

```
@SRR3882728.1.4052 TACAACCAC:DBR4KXP1:220:C27T3ACXX:5:1101:18655:7948 length=41
TTGAGGTAGTAGGTTGTATAGTTAACTCAAGTATACCTTTT
+SRR3882728.1.4052 TACAACCAC:DBR4KXP1:220:C27T3ACXX:5:1101:18655:7948 length=41
HHHHJJHIJGIIJHIJJJJJIJGJJJJIIIJGHIJJJJJJ
@SRR3882728.1.17301 TACAACCAC:DBR4KXP1:220:C27T3ACXX:5:1101:4014:27293 length=41
TTGAGGTAGTAGGTTGTATAGTTAACTCAAGTATACCTTTT
+SRR3882728.1.17301 TACAACCAC:DBR4KXP1:220:C27T3ACXX:5:1101:4014:27293 length=41
HHFHEGIIJIIIIHHIIIJJJJIJIIIIFGHGDHIHJIGIJJ
@SRR3882728.1.23800 TATAACCAA:DBR4KXP1:220:C27T3ACXX:5:1101:8037:36679 length=41
GCAACAATTCTACCTCAATTTGAGGTAGTAGGTTGTATAGT
+SRR3882728.1.23800 TATAACCAA:DBR4KXP1:220:C27T3ACXX:5:1101:8037:36679 length=41
HHHHJJJJJJJJJJJJJJJJJJJJEHHHIJJIIJIJIJJH
@SRR3882728.1.53673 TACAACCAC:DBR4KXP1:220:C27T3ACXX:5:1101:4134:79868 length=41
TTGAGGTAGTAGGTTGTATAGTTAACTCAAGTATACCTTTT
+SRR3882728.1.53673 TACAACCAC:DBR4KXP1:220:C27T3ACXX:5:1101:4134:79868 length=41
HHHHJJHHIIIHIIJJJJJJJJIJJJJJJGGFHJJJJJJJ
```

The first compressed FASTA file must correspond to the regulatory RNA sequences

```
>cel-let-7-5p
UGAGGUAGUAGGUUGUAUAGUU
>cel-lin-4-5p
UCCCUGAGACCUCAAGUGUGA
```

and the second FASTA file should correspond to all RNA sequences the user wants CLASH Analyst to query as the target sequences.

```
>C12C8.3a.1  (lin-41)
GCCACGGTTGGCGAACGGGTAAAAAGGAAGAGCCGATCGCCTCGTTACTCAAGGAAAAGG
CTCGACGTCGCTGGAGGAGGGAAAAGTTGGTTTTTTAGGATAAAAACCAACTTAAAGTAC
AAAAAGAGCCGGAAACGGCGGGAATGCGACGTTGGAAACGATCGTGATCTTGAATAAAG
ACTTGGAAAAAGTGAAATGGCGACCATCGTGCCATGCTCATTGGAGAAAGAAGAAGGAGC
ACCATCAGGACCTCGTCGGCTTCAAACTGAGATCGACGTGGACGCCAACGACAGCGGAAA
CGAGCTGTCGATGGGCGGAAGCAGCAGTGAAGGTGACTCAATGTCCCACCATCGCGGTGA
GCATTCACCAAACCATCATCATCAGGATAATCATCTTGGCTCGGGACCACCACCACCACAG
TTCACTGGCTCACTATTTGACACTCCACCGTCAATGATTCAGTCACCACAGCAGCAACCAC
AGTTCCAGTTCAACACTGGATTCGGACTCGGACTCCCGCAAGACTCCTTTCGGTGCTCAGT
CTGCTCCAAGAGCTCGACGATTGGAGTGCTGCCGTTCGTTTGTGCGCACAAAACGTGCCAA
TCATGCTACCAGATGACACCGTCCTCGTACGATCGACGGGCTTGCAAGCTGTGTGGTGCCG
TATCGACAGCCACCGCCAACTTTACATCGCAGATGTACCTGTCTCCGACGCTTCCATCACC
```

Each FASTA file should contain specific names, as these names will be used in all the available results.

CLASH read preprocessing

CLASH Analyst will perform all necessary steps to preprocess the raw reads (including adaptor/barcode sequence removal, filtering reads by quality, etc.) so that chimeras can be identified. The user can choose default settings or select specific settings according to how the CLASH libraries were prepared.

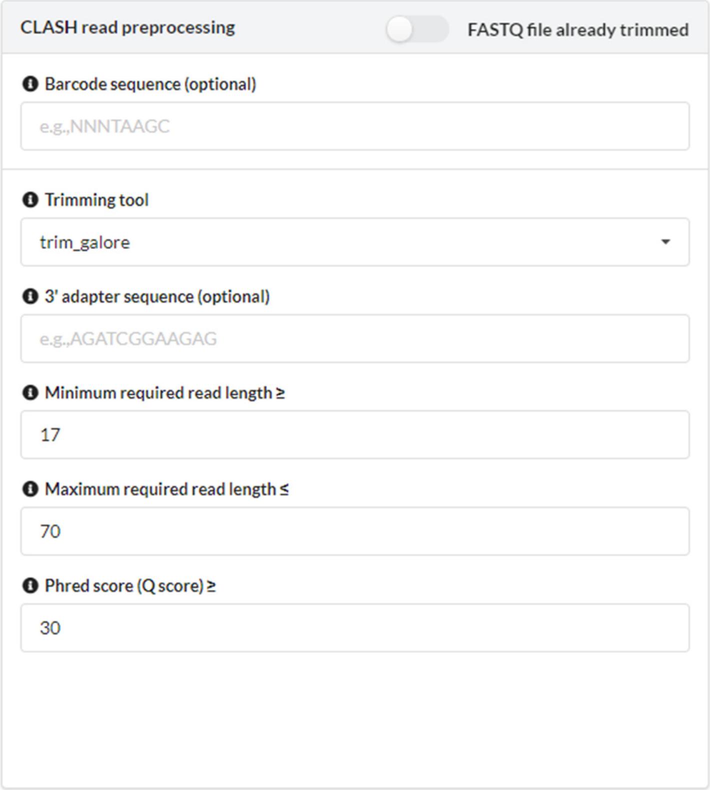

Users should provide adaptor information to allow precise trimming of adaptor sequences from the sequencing reads. Some library preparations include the ligation of a multiplexing barcode to the 5’ end of each RNA molecule. This sequence should be provided so that CLASH Analyst can trim it from reads before downstream analysis. Some library preparations may also include some number of random nucleotides during 5’ adaptor ligation to act as unique molecular identifiers (UMIs). The user should provide the number of random nucleotides included in the 5’ adaptor to allow CLASH Analyst to trim UMIs. For example, if the 5’ adaptor includes 6 random nucleotides, then the user should input NNNNNN as the 5’ barcode input. Similarly, 3’ adaptors sequence can be provided for precise trimming. Since Trim galore is capable of detecting 3’ adaptor sequences without prompting, Trim galore is chosen as the default trimming tool (1). If the user chooses this tool then 3’ adaptor sequence input is optional (although providing the sequence can help). The user can also select from two other tools, flexbar or fastx, to perform adaptor trimming (2,3). Based on how molecules were collected during library construction, the user should have an expectation of the length of reads following adaptor trimming and select/filter only reads of a certain length range for analyses. For example, if RNA molecules between 17-70nt long were selected and ligated to adaptors, then the trimmed reads should be 17-70nt in length. The user can adjust the range of RNA lengths that will be analyzed. Finally, the user can filter reads that do not meet a quality threshold encoded in the FASTQ file, which is called the Phred score. Phred scores are assigned during sequencing based on the probability of errant base calling. The default threshold for the Phred score is 30. A Phred score of 30, 40, or 50 for a particular base corresponds to a 99.9%, 99.99%, or 99.999% likelihood that the called base is accurate, respectively. CLASH Analyst can also accept data that has been pre-processed prior to being uploaded if the user wishes to perform these steps themselves.

Identify Chimeras

Once data is pre-processed CLASH Analyst will identify chimeric reads using default or user-defined settings. The user can choose between 3 different algorithms to assign chimeric reads: CLAN (4), Hyb (5), and piRTarBase (6) (see figure below). These algorithms differ on how they search for chimeric reads, resulting in some differences in the set of interactions they each identify. The default searching tool in CLASH Analyst is CLAN.

If the user chooses to use CLAN, they can use default settings or select the minimum length of each fragment within a chimera read for analyses. Additionally, the user can specify how much overlap between the two fragments is allowed. If the default value of 4 is selected, then 4nt would be allowed to map to each fragment simultaneously. The user can also specify how many hits will be allowed for each fragment, as each fragment could match multiple user-input FASTA sequences.

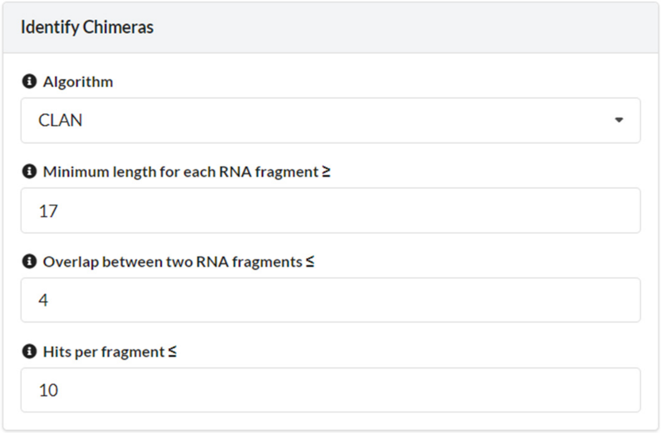

If the user chooses to use Hyb, the overlap allowance and multiple hit allowance must be specified as with CLAN, as well as a fragment selection threshold, which is the same as the e-value used in BLAST (7). This value represents the number of hits expected by chance for a given mapped sequence to be found elsewhere with the same mapping quality. The

default value of 0.1 corresponds to a tolerance of up to a 10% chance of a given mapped read to be found to map with the same quality by chance.

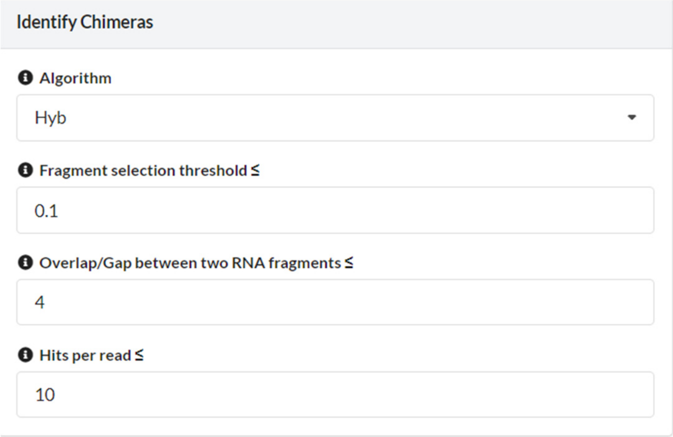

If the user chooses to use piRTarBase, then they should specify the maximum number of mismatches allowed in each fragment within a chimera – both the regulatory RNA fragment and the target RNA fragment. Choosing the default of 0 for each will result in chimeric reads only being identified if they match both a regulatory RNA and remaining read sequence with 100% sequence identity. piRTarBase requires the full regulatory RNA sequence to be present in a given read, so users can specify the required length of the remaining sequence. Like with CLAN, the user can also specify the hit allowance.

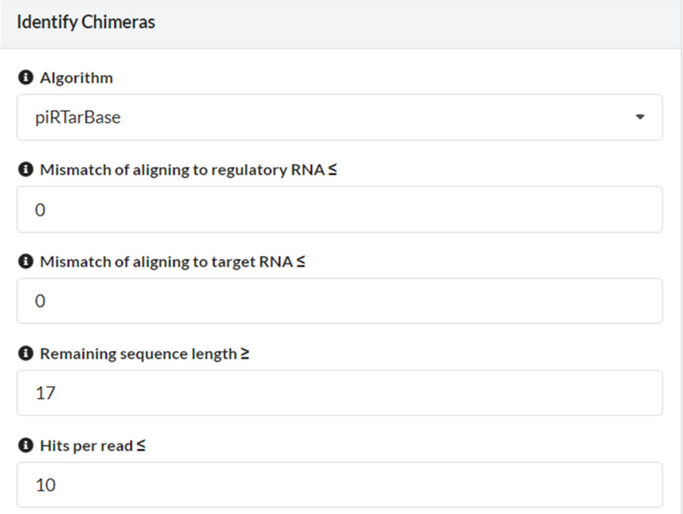

We recommend users to first analyze their data with CLAN if they are unsure of which algorithm to choose, as CLAN will perform the least stringent analysis. Then, to further refine the results, users can choose to re-run their data using Hyb or piRTarBase.

User information

Once the user has uploaded all data and selected all necessary settings, they can submit the analysis job by pressing submit at the bottom of the page. A Job ID and a website link are provided for the users to check the status and examine the results.

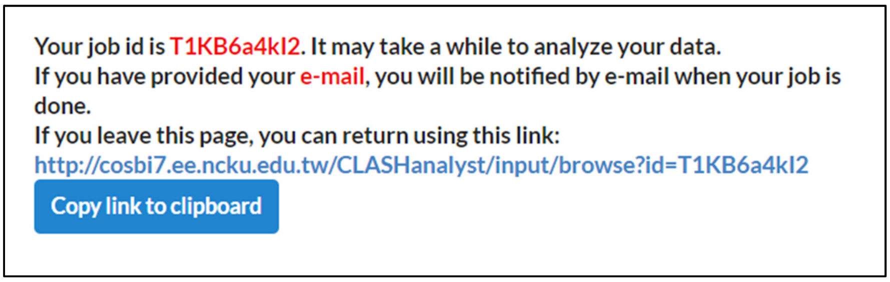

The analysis can typically take a few hours to run (sometimes a day for larger datasets), so we have provided an option to input an email address for the user to be notified once the analysis is complete. This is an optional step and the email address will be used only to notify and send the user a link to the results page.

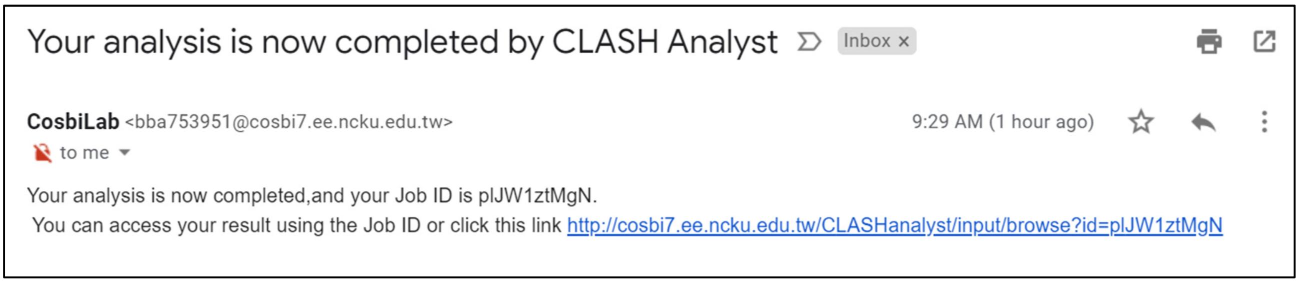

Chimeric Reads Output

Once a user receives an email with a link to the results page or has checked the link after the run is complete, then they can examine the results within CLASH Analyst. A summary of

the analysis is first displayed in two tables. The first shows all the user-specified settings that were used to perform the analysis while the second shows the number of reads provided by the user, the number of reads and unique reads after trimming the adaptors, the number of those trimmed reads that were identified as chimeric, and the number of RNA-RNA interactions identified. The user can also download the complete results in .csv format for additional analyses, if needed.

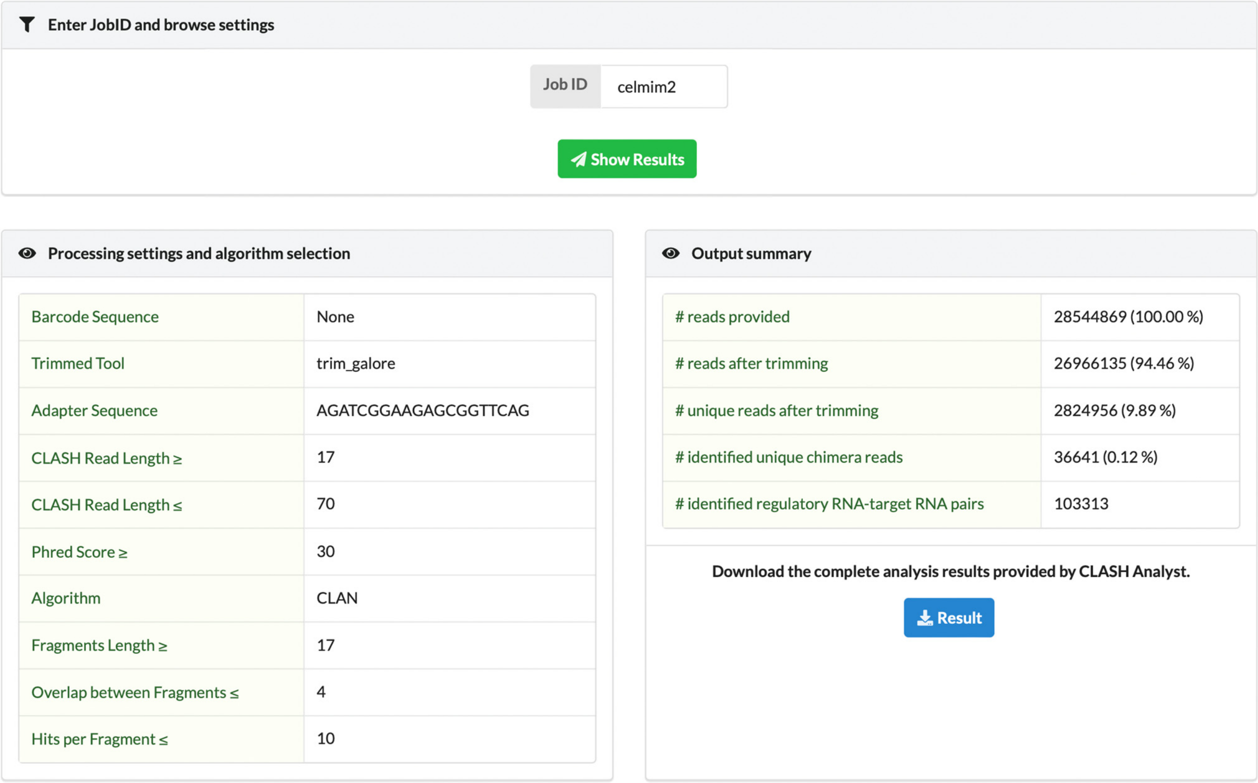

Barcode Sequence	None
Trimmed Tool	trim_galore
Adapter Sequence	AGATCGGAAGAGCGGTTCAG
CLASH Read Length ≥	17
CLASH Read Length ≤	70
Phred Score ≥	30
Algorithm	CLAN
Fragments Length ≥	17
Overlap between Fragments ≤	4
Hits per Fragment ≤	10

# reads provided	28544869 (100.00 %)
# reads after trimming	26966135 (94.46 %)
# unique reads after trimming	2824956 (9.89 %)
# identified unique chimera reads	36641 (0.12 %)
# identified regulatory RNA-target RNA pairs	103313

The user can select how the results should be displayed, either by identified regulatory RNAs within the chimera, by identified target RNAs within the chimera, or by identified RNA-RNA pairs. The browsable and searchable table containing the remainder of the output will depend on which browse mode was selected and will be discussed separately here.

<u>Browse modes</u>

1. Browse by Regulatory RNA Name

The browsable and searchable output table will contain 3 columns: a column corresponding to regulatory RNA names, a column displaying the number of the identified target RNAs for each regulatory RNA, and an option to show more details about the targets for each regulatory RNA (see figure below).

Browse Result

Browse by | Regulatory RNA name | Select

Show 10 entries Search:

Regulatory RNA Name	# Identified Targets	Target RNA Details
cel-let-7-5p	8	show target names
cel-lin-4-5p	2	show target names

Showing 1 to 2 of 2 entries Previous 1 Next

When clicking “show target names”, it will reveal all target RNAs identified within chimeras containing the selected regulatory RNA in a tabular format. Within the table, users can find the number of chimeric reads representing the number of unique chimeras containing that RNA-RNA interaction. Additionally, any of the regulatory RNA-target RNA pairs can be further examined by clicking “show interaction” (see figure below).

Target Detail

The regulatory RNA cel-let-7-5p has 8 target RNAs.

Show 10 entries Search:

Target RNA Name	# Unique Chimera Reads	Interaction Details
C12C8.3a.1 (lin-41)	14	show interaction
C12C8.3a.2 (lin-41)	14	show interaction
C12C8.3a.3 (lin-41)	14	show interaction
C12C8.3b.1 (lin-41)	14	show interaction
C12C8.3b.2 (lin-41)	14	show interaction
C12C8.3b.3 (lin-41)	14	show interaction
T25C12.1a.1 (lin-14)	4	show interaction
T25C12.1b.1 (lin-14)	4	show interaction

Showing 1 to 8 of 8 entries Previous 1 Next

This will reveal a graphic showing the interactions between target RNA and regulatory RNA, as well as a table that shows details of each identified interactions (see figure below).

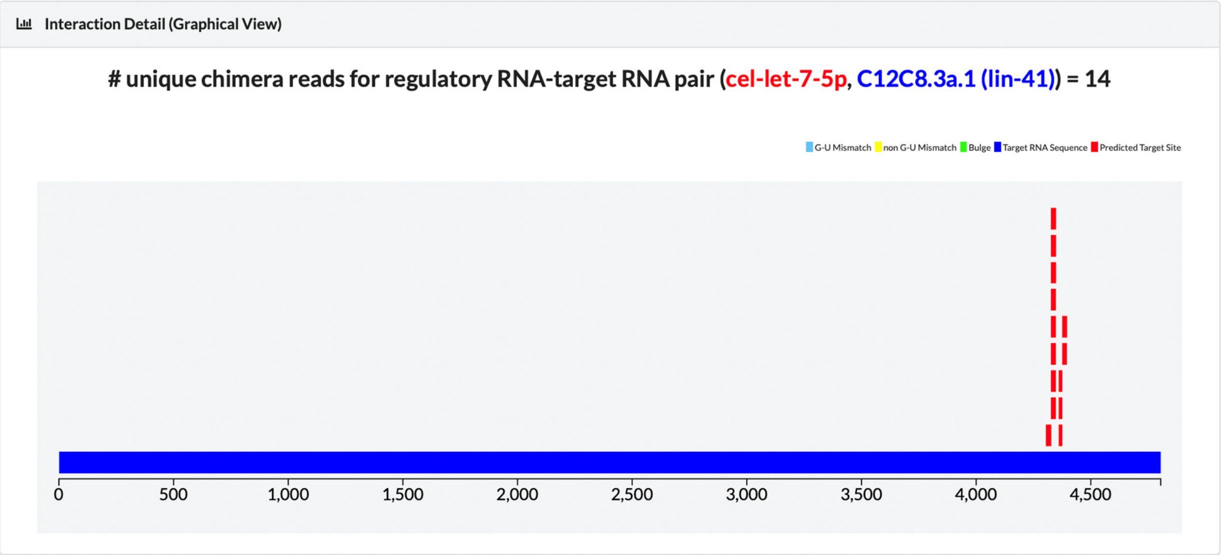

Interaction Detail (Table View)

unique chimera reads for regulatory RNA-target RNA pair (cel-let-7-5p, C12C8.3a.1 (lin-41)) = 14

G-U Mismatch non G-U Mismatch Bulge

Show 10 entries Search:

CLASH Read ID	Read Count	Regulatory RNA Name	Regulatory RNA Length	CLASH Identified Region	Predicted Target Site	RNAup Score	Pairing (Top:Target,Bottom:Regulator)
hybrid4	19	cel-let-7-5p	22	4358-4376	4360-4376	-7.1	5'AAUAUCGCAAUC-----CCUUU3'<br>3'UUGAUAUGUUGGAUGAUGGAGU5'
hybrid5	7	cel-let-7-5p	22	4322-4339	4326-4348	-19.15	5'UUUUAUACAACCGUUCUACACUC3'<br>3'UUGAUAUGUUGG-AUGAUGGAGU5'
hybrid6	4	cel-let-7-5p	22	4326-4344	4326-4349	-20.73	5'UUUUAUACAACCGUUCUACACUCA3'<br>3'UU--GAUAUGUUGGAUGAUGGAGU5'
hybrid7	6	cel-let-7-5p	22	4370-4390	4375-4396	-21.93	5'UUUUAUACAACCAUUCUGCCUC3'<br>3'UUGAUAUGUUGGAUGAUGGAGU5'
hybrid10	3	cel-let-7-5p	22	4322-4341	4326-4348	-19.15	5'UUUUAUACAACCGUUCUACACUC3'<br>3'UUGAUAUGUUGG-AUGAUGGAGU5'
hybrid12	20	cel-let-7-5p	22	4327-4347	4326-4349	-20.73	5'UUUUAUACAACCGUUCUACACUCA3'<br>3'UUGAUAUGUUGG-AUGAUG-GAGU5'
hybrid15	17	cel-let-7-5p	22	4326-4346	4326-4349	-20.73	5'UUUUAUACAACCGUUCUACACUCA3'<br>3'UU--GAUAUGUUGGAUGAUGGAGU5'
hybrid18	1	cel-let-7-5p	22	4363-4380	4360-4376	-7.1	5'AAUAUCGCAAUC-----CCUUU3'<br>3'UUGAUAUGUUGGAUGAUGGAGU5'
hybrid19	17	cel-let-7-5p	22	4312-4329	4304-4328	-10.24	5'AUUGCACCAACUCAAGUAUACCUUU3'<br>3'UUGAUAUGUUGG---AUGAUGGAGU5'
hybrid20	9	cel-let-7-5p	22	4322-4340	4326-4348	-19.15	5'UUUUAUACAACCGUUCUACACUC3'<br>3'UUGAUAUGUUGG-AUGAUGGAGU5'

Showing 1 to 10 of 14 entries Previous 1 2 Next

This table shows information about each hybrid, including the number of reads, the thermodynamic favorability of the regulatory RNA and target RNA fragments identified in the hybrid, where the fragments are mapped in the hybrid, and the base pairing between the identified fragments.

2. Browse by Target RNA Name

The output table will contain 4 columns: a column corresponding to target RNA name, a column displaying the number of unique chimeras that contained that target RNA, a column displaying the number of unique regulatory RNAs found to pair with that target, and an option to show interactions involving each target RNA.

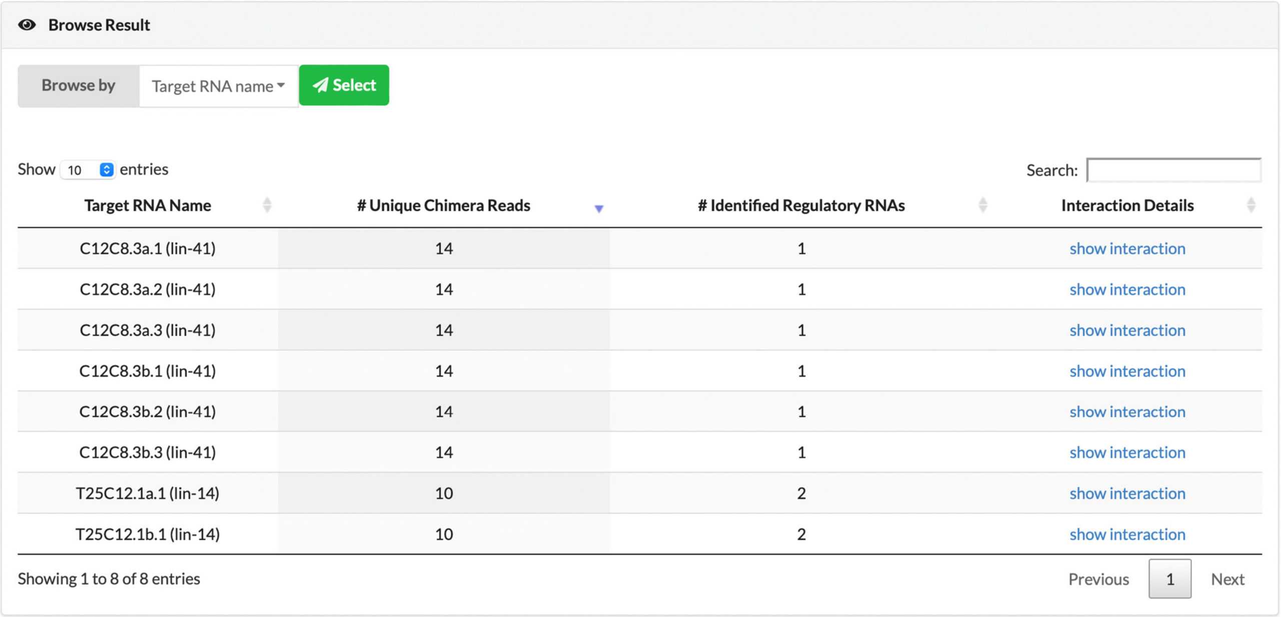

Target RNA Name	# Unique Chimera Reads	# Identified Regulatory RNAs	Interaction Details
C12C8.3a.1 (lin-41)	14	1	show interaction
C12C8.3a.2 (lin-41)	14	1	show interaction
C12C8.3a.3 (lin-41)	14	1	show interaction
C12C8.3b.1 (lin-41)	14	1	show interaction
C12C8.3b.2 (lin-41)	14	1	show interaction
C12C8.3b.3 (lin-41)	14	1	show interaction
T25C12.1a.1 (lin-14)	10	2	show interaction
T25C12.1b.1 (lin-14)	10	2	show interaction

By clicking “show interaction” the user can view a detailed graphic and table outlining all interactions involving that target RNA (as shown in the previous browse mode).

3. Browse by RNA-RNA Pair

Users can also browse by RNA-RNA Pair. The results can either be sorted according to read count or according to the binding energy (represented as RNAup score) (8). The output table will have rows corresponding to each unique chimera, and will show information about each hybrid, including the read count, the thermodynamic favorability (represented by RNAup score) of the regulatory RNA and target RNA fragments identified in the hybrid, where the fragments are mapped in the hybrid, and the base pairing between the identified fragments. The webpage will only show the first 10,000 results. To view the complete results, the user can download the result table as discussed above.

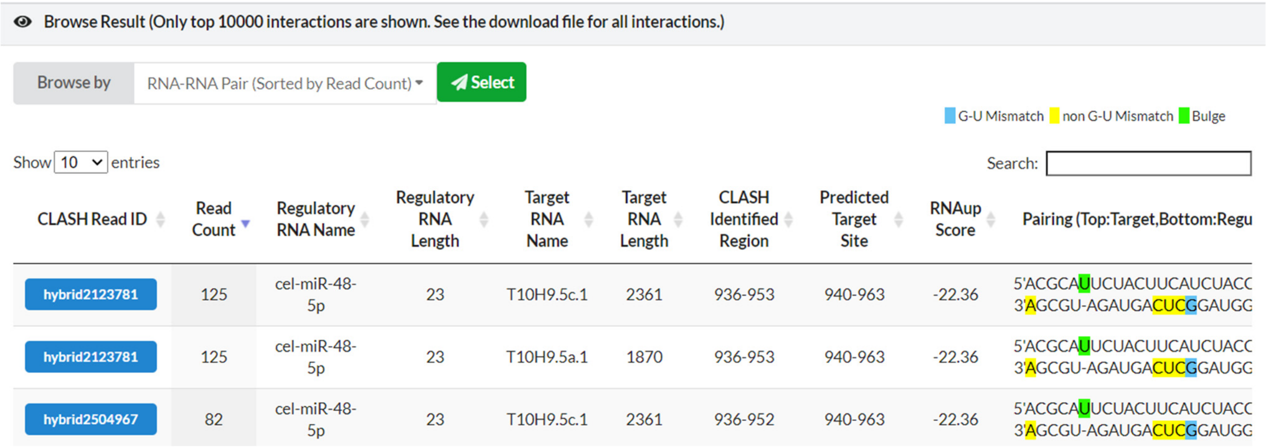
Browse Result (Only top 10000 interactions are shown. See the download file for all interactions.)

Browse by | RNA-RNA Pair (Sorted by Read Count) | Select

G-U Mismatch | non G-U Mismatch | Bulge

Show 10 entries | Search:

CLASH Read ID	Read Count	Regulatory RNA Name	Regulatory RNA Length	Target RNA Name	Target RNA Length	CLASH Identified Region	Predicted Target Site	RNAup Score	Pairing (Top:Target,Bottom:Regu
hybrid2123781	125	cel-miR-48-5p	23	T10H9.5c.1	2361	936-953	940-963	-22.36	5'ACGCAUUCUACUUCAUCUACC<br>3'AGCGU-AGAUGACUCGGAUGG
hybrid2123781	125	cel-miR-48-5p	23	T10H9.5a.1	1870	936-953	940-963	-22.36	5'ACGCAUUCUACUUCAUCUACC<br>3'AGCGU-AGAUGACUCGGAUGG
hybrid2504967	82	cel-miR-48-5p	23	T10H9.5c.1	2361	936-952	940-963	-22.36	5'ACGCAUUCUACUUCAUCUACC<br>3'AGCGU-AGAUGACUCGGAUGG

CLASH Analyst Webserver construction

The CLASH Analyst analysis pipeline behind the web interface is implemented using the awk, bash and Python scripts. The web interface of CLASH Analyst was constructed using Django, a Python web framework that encourages rapid web development. All figures in CLASH Analyst were generated using D3.js, a JavaScript library which provides powerful visualization components. All tables in CLASH Analyst were generated using DataTables, a table enhancing plug-in for the jQuery Javascript library which adds sorting, paging and

filtering abilities to plain HTML tables with minimal effort. CLASH Analyst is available at https://cosbi7.ee.ncku.edu.tw/CLASHanalyst/ (main site) or https://cosbi.ee.ncku.edu.tw/CLASHanalyst/ (backup site).

Browser compatibility

OS	Version	Chrome	Firefox	MicrosoftEdge	Safari
Linux	CentOS 6	n/a	84.0	n/a	n/a
MacOS	HighSierra	87.0	n/a	n/a	11.1
Windows	10	87.0	84.0	87.0	n/a

References cited in supplementary information

1. Martin M. Cutadapt removes adapter sequences from high-throughput sequencing reads. EMBnet.journal [Internet]. 2011 May 2;17(1):10. Available from: http://journal.embnet.org/index.php/embnetjournal/article/view/200
2. Dodt M, Roehr JT, Ahmed R, Dieterich C. FLEXBAR-flexible barcode and adapter processing for next-generation sequencing platforms. Biology (Basel). 2012;1(3):895–905.
3. Lab H. FASTX-Toolkit (RRID:SCR_005534) [Internet]. Available from:

http://hannonlab.cshl.edu/fastx_toolkit/

4. Zhong C, Zhang S. Accurate and Efficient Mapping of the Cross-Linked microRNA-mRNA Duplex Reads. iScience [Internet]. 2019;18:11–9. Available from: https://doi.org/10.1016/j.isci.2019.05.038

5. Travis AJ, Moody J, Helwak A, Tollervey D, Kudla G. Hyb: A bioinformatics pipeline for the analysis of CLASH (crosslinking, ligation and sequencing of hybrids) data. Methods [Internet]. 2014;65(3):263–73. Available from: http://dx.doi.org/10.1016/j.ymeth.2013.10.015

6. Wu WS, Brown JS, Chen T Te, Chu YH, Huang WC, Tu S, et al. PiRTarBase: A database of piRNA targeting sites and their roles in gene regulation. Nucleic Acids Res. 2019;47(D1):D181–7.

7. Altschul SF, Gish W. Local Alignment Statistics. Seq Comp. 2008;266:119–47.

8. Gruber AR, Bernhart SH, Lorenz R. The ViennaRNA Web Services. In: Picardi E, editor. RNA Bioinformatics [Internet]. New York, NY: Springer New York; 2015. p. 307–26. Available from: https://doi.org/10.1007/978-1-4939-2291-8_19
